# External Knee Adduction and Flexion Moments during Gait and Medial Tibiofemoral Disease Progression in Knee Osteoarthritis

**DOI:** 10.1016/j.joca.2015.02.005

**Published:** 2015-02-10

**Authors:** Alison H. Chang, Kirsten C. Moisio, Joan S. Chmiel, Felix Eckstein, Ali Guermazi, Pottumarthi V. Prasad, Yunhui Zhang, Orit Almagor, Laura Belisle, Karen Hayes, Leena Sharma

**Affiliations:** 1 Department of Physical Therapy and Human Movement Sciences, Feinberg School of Medicine, Northwestern University, Chicago, IL, USA; 2 Department of Preventive Medicine, Feinberg School of Medicine, Northwestern University, Chicago, IL, USA; 3 Institute of Anatomy, Paracelsus Medical University Salzburg & Nuremberg, Salzburg, Austria; 4 Department of Radiology, Boston University School of Medicine, Boston, MA, USA; 5 Department of Radiology, NorthShore University HealthSystem, Evanston, IL, USA; 6 Department of Medicine, Feinberg School of Medicine, Northwestern University, Chicago, IL, USA

## Abstract

**Objective:**

Test the hypothesis that greater baseline peak external knee adduction moment (KAM), KAM impulse, and peak external knee flexion moment (KFM) during the stance phase of gait are associated with baseline-to-2-year medial tibiofemoral cartilage damage and bone marrow lesion progression, and cartilage thickness loss.

**Methods:**

Participants all had knee OA in at least one knee. Baseline peak KAM, KAM impulse, and peak KFM (normalized to body weight and height) were captured and computed using a motion analysis system and 6 force plates. Participants underwent MRI of both knees at baseline and two years later. To assess the association between baseline moments and baseline-to-2-year semiquantitative cartilage damage and bone marrow lesion progression and quantitative cartilage thickness loss, we used logistic regression with generalized estimating equations (GEE), adjusting for gait speed, age, gender, disease severity, knee pain severity, and medication use.

**Results:**

The sample consisted of 391 knees (204 persons): mean age 64.2 years (SD 10.0); BMI 28.4 kg/m^2^ (5.7); 156 (76.5%) women. Greater baseline peak KAM and KAM impulse were each associated with worsening of medial bone marrow lesions, but not cartilage damage. Higher baseline KAM impulse was associated with 2-year medial cartilage thickness loss assessed both as % loss and as a threshold of loss, whereas peak KAM was related only to % loss. There was no relationship between baseline peak KFM and any medial disease progression outcome measures.

**Conclusion:**

Findings support targeting KAM parameters in an effort to delay medial OA disease progression.

## Introduction

Osteoarthritis (OA) is a leading contributor to chronic disability (1). Twenty-three percent of U.S. adults report doctor-diagnosed arthritis and 10% have arthritis-related activity limitations (2). OA is the most common form of arthritis, frequently affecting the knee. The impact of knee OA in the U.S. is likely to increase due to the aging population, obesity epidemic, and paucity of disease-modifying treatment. It is well accepted that an abnormal knee local mechanical environment can contribute to joint damage. Change in medial-to-lateral tibiofemoral load distribution and greater medial load are theorized to increase the risk of medial knee OA disease progression (3).

Instrumented force-measuring knee implantation is the current gold standard method for measurement of medial knee load, but it is invasive and impractical. Knee load cannot be directly measured *in vivo* noninvasively. The external knee adduction moment (KAM) during the stance phase of gait has been characterized both as a determinant and a surrogate for dynamic medial knee load (3,4). KAM reflects the medial-to-lateral joint load distribution (5) and has been associated with lower limb varus alignment (6), medial OA disease severity (7), and medial-to-lateral bone mineral density ratio (8). Efforts have been directed toward developing and testing interventions that lower KAM with the ultimate goal of modifying disease course in medial tibiofemoral OA (9,10). However, longitudinal evidence of an association between baseline KAM and subsequent medial disease progression comes from only a few studies with inconsistent findings (11,12,13).

Peak KAM during the stance phase potentially captures maximal medial joint load experienced at any one instant of time. KAM impulse is the time integral of KAM over the stance phase. By incorporating both load magnitude *and* duration, KAM impulse may provide a cumulative measure of KAM sustained during each step of walking. There is a theoretical rationale to support a role for both of these parameters in disease progression. Studies in recent years suggest that a reduction in KAM may be accompanied by a deleterious increase in the external knee flexion moment (KFM) (14,15). However, whether KFM plays a role in knee OA disease progression in OA knees is unclear.

The objective of this study was to evaluate the association between baseline KAM and KFM parameters and subsequent medial tibiofemoral OA disease progression over 2 years. We hypothesized that in persons with knee OA, greater baseline peak KAM, KAM impulse, and peak KFM (each normalized to body weight and height) during the stance phase of gait are each associated with baseline-to-2-year worsening of medial tibiofemoral cartilage damage and bone marrow lesions, and with quantitatively measured cartilage thickness loss.

## Methods

### Sample

In this prospective, longitudinal, observational cohort study of knee OA, the MAK-3 Study (Mechanical Factors in Arthritis of the Knee-Study 3), participants were recruited from the community using advertising in periodicals, neighborhood organizations, letters to the Buehler Center on Aging, Health, and Society registry at Northwestern University, and via medical center referrals. Inclusion criteria were: definite tibiofemoral osteophyte presence [Kellgren/Lawrence (K/L) radiographic grade ≥ 2] in one or both knees; and Likert category of at least “a little difficulty” for 2 or more items in the WOMAC physical function scale. Exclusion criteria were: corticosteroid injection within previous 3 months; avascular necrosis, inflammatory arthritis, periarticular fracture, Paget's disease, villonodular synovitis, joint infection, ochronosis, neuropathic arthropathy, acromegaly, hemochromatosis, gout, pseudogout, osteopetrosis, or meniscectomy; or MRI exclusions. Approval was obtained from the Institutional Review Boards of Northwestern University and NorthShore University HealthSystem Evanston Hospital. All participants provided written consent.

### Quantitative gait analysis

Kinematic data were collected at 120 Hz, using an 8-camera, Eagle Digital Real-Time motion measurement system from Motion Analysis Corporation (MAC). At a sampling rate of 960 Hz, ground reaction forces and moments were measured with 6 AMTI (Advanced Mechanical Technology Inc., Watertown, MA, USA) force platforms embedded flush with the floor as participants walked along a 10.7 × 1.2 meter walkway. An experienced technician placed external passive reflective markers, using the modified Helen Hayes full-body marker set (16) (bilaterally on acromion process tip, lateral humeral epicondyle, between radius and ulna styloids, anterior superior iliac spine, superior sacrum at L5/sacral interface, lower thigh, along flexion/extension rotation axis at lateral femoral condyle, lower leg, along flexion/extension rotation axis at lateral malleolus, posterior calcaneus, foot center between 2^nd^ and 3^rd^ metatarsals). To closely match usual daily walking, each participant wore his/her own comfortable athletic or walking shoes and walked at a self-selected comfortable speed without using assistive devices (no participant habitually used assistive devices). A minimum of five trials having clean foot strikes on the force platforms for the left and right feet were acquired, with rest between trials. OrthoTrak gait analysis software (MAC) was used to calculate 3-D joint angles, moments, and temporal-spatial parameters. Inverse dynamics were used to compute 3-D external joint moments. Baseline predictors of peak KAM (% body weight*height), KAM impulse – the area under the KAM-time curve (seconds*% body weight*height), and KFM (% body weight*height) were calculated using custom Matlab programs. Gait speed was measured within the quantitative gait analysis; the 5-trial average was used.

While KAM normalization is widely accepted and established, to address the possibility that the absolute (i.e., non-normalized) KAM parameter values differed in pattern of association with the outcomes, we evaluated the correlation between normalized and non-normalized values, and, in sensitivity analyses, the association between non-normalized KAM parameters and outcomes.

### MRI acquisition and semi-quantitative assessment of cartilage damage and bone marrow lesion progression

At baseline and 2-year follow-up, magnetic resonance images (MRI) of both knees were obtained in all participants, using a commercial knee coil and 1 of 2 whole-body scanners, 3T Verio or 1.5T Avanto (both Siemens Healthcare, Erlangen, Germany); the same scanner was used at both evaluations. The protocol included coronal T1-weighted spin-echo (SE) [TR/TE/FOV/Matrix/Slice thickness = 3 s/20 ms/14 cm, 256×256, 3 mm at 3T; TR/TE/FOV/Matrix/Slice thickness = 3 s/18 ms/14 cm, 256×256, 3 mm at 1.5T], and sagittal axial, and coronal fat-suppressed proton density-weighted turbo spin echo sequences [TR/TE/Turbo Factor/FOV/Matrix/Slice thickness = 500 ms/11 ms/7/12 cm, 320×320, 3 mm at 3T; TR/TE/Turbo Factor/FOV/Matrix/Slice thickness = 600 ms/11 ms/7/12 cm, 320×320, 3 mm at 1.5T].

Following a detailed reading protocol, each knee was scored using the Whole-Organ MRI Score (WORMS) method (17), by one of two expert musculoskeletal radiologists. Baseline and 2-year scans were evaluated as pairs, with known chronology as suggested for longitudinal studies in knee OA (18), but blinded to all other data. Two medial weightbearing femoral condylar subregions (central and posterior) and 3 medial tibial plateau subregions (anterior, central, and posterior) were each scored separately for cartilage morphology and bone marrow lesions. At each subregion, cartilage morphology was scored: 0 (normal thickness and signal); 1 (normal thickness, increased signal on T2-weighted images); 2 (solitary, focal, partial or full-thickness defect ≤ 1 mm in width); 3 (multiple areas of partial-thickness loss or grade 2 lesion > 1 mm, with areas of preserved thickness); 4 (diffuse, > 75%, partial-thickness loss); 5 (multiple areas of full-thickness loss, or full-thickness lesion > 1 mm, with areas of partial-thickness loss); and 6 (diffuse, > 75%, full-thickness loss). Subchondral bone marrow lesions were scored: 0 (normal); 1 (mild, < 25% of region); 2 (moderate, 25-50% of region); and 3 (severe, > 50% of region). In a previous study, the inter-rater intra-class correlation coefficients (ICCs) (unspecified model) for these same readers were 0.98 and 0.90 for medial cartilage morphology and bone marrow lesions respectively (17).

Baseline-to-2-year progression of cartilage damage and bone marrow lesions in the medial tibiofemoral compartment were each defined as a full-grade score worsening in any of the 5 medial femoral and tibial subregions. Medial femoral surface progression was defined as score worsening in either of the 2 femoral subregions, and tibial surface progression as worsening in any of the 3 tibial subregions.

### Quantitative measurement of cartilage thickness loss

For the quantitative cartilage measurement, coronal spoiled gradient echo sequences with water excitation were acquired, with a slice thickness of 1.5 mm and an in-plane resolution of 0.31 mm (field of view 16 cm, 512 _ 512–pixel matrix, number of excitations 1). The repetition time, echo time, and flip angle, respectively, were 18.6 msec/9.3 msec/15° on the 1.5T, and 12.2 msec/5.8 msec/9° on the 3T scanner; baseline and follow-up acquisitions were always done using the same magnet. The total area of subchondral bone and the area of the cartilage surface were segmented in the medial tibial surfaces, and in the weight-bearing portion of the medial femoral condyles using proprietary software (Chondrometrics, Ainring, Germany) (19–22).

Average thickness of cartilage, including areas of denuded subchondral bone as 0 mm, was quantified in baseline and 2-year images with chronology known (18). Using the same methodology, cartilage thickness precision error (coefficient of variation [CV] for 2 acquisitions with repositioning) was 2.1% for the medial tibia and 3.0% for the medial weightbearing femur (19). The regions of interest (ROI) in this study were the entire medial tibial and central weightbearing femoral surfaces; external, central, and posterior tibial subregions and external and central femoral subregions (22), since greater 12-month cartilage thinning and standardized response means were observed in these than other subregions (23). Disease progression outcome in each ROI was analyzed as a continuous outcome variable expressed as % cartilage loss over the baseline-to-2 year follow-up period, and secondarily as a dichotomous variable defined as baseline-to-2-year cartilage thickness loss ≥ 5% (i.e., approximately twice the CV, as previously defined (19), a threshold that is unlikely to reflect measurement error).

### Assessment of disease severity, alignment, knee pain, and medication use

All participants underwent bilateral, anteroposterior, weightbearing knee radiographs at baseline in the semiflexed position with fluoroscopic confirmation of superimposition of the anterior and posterior tibial plateau lines and centering of the tibial spines within the femoral notch (24). Disease severity was assessed using the K/L system, 0 (normal), 1 (possible osteophytes), 2 (definite osteophytes, with possible joint space narrowing), 3 (moderate osteophytes with definite joint space narrowing, some sclerosis, and possible attrition), and 4 (large osteophytes with marked joint space narrowing, severe sclerosis, and definite attrition) (25). To assess knee alignment, a single anteroposterior radiograph of both limbs was obtained using a 1.3 by 0.4-meter graduated-grid cassette. All radiographs were obtained in the same unit by two trained technicians. Alignment was measured as the angle formed by the intersection of the line connecting the centers of the femoral head and intercondylar notch with the line connecting the centers of the surface of the ankle talus and tips of the tibial spines. Alignment was recorded as negative for the varus direction, 0° for neutral, and positive for valgus. Image analysis (26) was completed by one of the three trained readers using a customized program (Surveyor 3; OAISYS Inc, Kingston, Ontario, Canada), blinded to all other data. In a reliability study of 200 full-limb pairs assessed by these readers, the inter- and intra-reader ICCs were 0.95 and 0.96 (27).

Knee pain severity was measured using the Intermittent and Constant Osteoarthritis Pain (ICOAP), a valid and reliable multidimensional measure designed to comprehensively evaluate the pain experience in knee or hip OA (28,29). Medication use was defined as a yes answer to: During the past 30 days, have you used any of the following medications for joint pain or arthritis on most days? (for at least one category among acetaminophen, non-prescription NSAIDs, prescription NSAIDs, and prescription pain medications).

### Statistical analysis

To assess the relationships between baseline peak KAM, KAM impulse, and peak KFM (each as a continuous variable) and subsequent cartilage damage and bone marrow lesion progression (dichotomous outcomes) in the medial tibiofemoral compartment and at femoral and tibial surfaces, we used logistic regression with generalized estimating equations (GEE) to account for the correlation between the 2 limbs of each individual, adjusting for gait speed, age, gender, disease severity, knee pain severity, and medication use. Results are reported as odds ratios (ORs) and 95% confidence intervals (CIs). Similarly, linear and logistic regression models with GEE methods were used to assess the relationships between baseline peak KAM, KAM impulse, and peak KFM and quantitative cartilage thickness loss outcomes. In sensitivity analyses, we used the same models with non-normalized KAM parameters.

## Results

Among 250 participants, 212 completed the 2-year follow-up visit. Reasons for not completing included: participant not reachable (n=12); serious medical condition (n=6); too busy (n=5); work (n=5); other (n=10). An additional 8 participants developed a contraindication or declined the follow-up MRI. Among the remaining 204 participants (408 knees), 14 knees were excluded due to a total knee replacement and 3 knees had technical image problems, leaving the final analysis sample of 391 knees from 204 participants. The mean age of the 204 participants was 64.2 (SD 10.0) years, mean BMI was 28.4 (5.7) kg/m^2^, and 156 (76.5%) were women. Mean knee mechanical axis was -1.0 (4.0) degrees (i.e., in the varus direction). The K/L grade distribution was: grade 0, 17 knees (4.3%); grade 1, 72 (18.4%); grade 2, 186 (47.6%); grade 3, 56 (14.3%); and grade 4, 60 knees (15.4%). Mean gait speed was 1.2 (0.2) m/s. Mean pain severity score was 9.24 (7.58) and 92 persons (45.1%) were taking medication on most of the past 30 days. Mean baseline peak KAM, KAM impulse, peak KFM were 1.67 (0.85) % body weight*height, 0.60 (0.44) seconds*% body weight*height, and 2.09 (0.85) % body weight*height respectively. Participants who did not complete the follow-up did not differ from completers in KAM, KAM impulse, gender, BMI, knee alignment, K/L grade, and medication use. The non-completers, however, differed slightly in age [68.1 (11.1) years, p=0.03], gait speed [1.1 (0.2) m/s, p=0.002], peak KFM [1.78 (0.78) % body weight*height, p=0.001], and knee pain severity [11.74 (7.76), p=0.02].

Table 1 provides the mean baseline peak KAM, KAM impulse, and peak KFM for knees without vs. with baseline-to-2-year cartilage damage progression, and for knees without vs. with bone marrow lesion progression. Table 2 shows the pairwise Spearman correlations for variables at baseline (right knee only). KAM and KAM impulse were highly correlated with each other and were each highly correlated with varus alignment. Because of this, and because it very likely falls in the casual KAM/disease progression pathway, varus alignment was not a covariable for inclusion in multivariable models. As in Table 3, greater baseline peak KAM was significantly associated with bone marrow lesion progression at the medial tibial surface, and KAM impulse with bone marrow lesion progression in the medial tibiofemoral compartment and specifically at the tibial surface. There was no evidence of an association between baseline peak KAM or KAM impulse and cartilage damage progression (Table 3). Baseline peak KFM was not associated with cartilage damage (e.g., for medial tibiofemoral compartment, adjusted OR 0.96, 95% CI: 0.65, 1.42) or bone marrow lesion progression (e.g., for medial tibiofemoral compartment, adjusted OR 1.19, 95% CI: 0.90, 1.58).

In the assessment of cartilage thickness change, 6 additional knees were excluded due to image technical problems, leaving 385 knees for analysis. As in Table 4, greater baseline peak KAM and KAM impulse were each significantly associated with greater baseline-to-2-year % cartilage thickness loss as a continuous variable, at the medial tibial surface, external and central tibial subregions, central femoral weightbearing surface, and central femoral subregion. Table 5 shows mean baseline peak KAM, KAM impulse, and peak KFM among knees without and with ≥ 5% cartilage thickness loss. In analyses of the secondary outcome, KAM impulse was significantly associated with ≥ 5% cartilage thickness loss at the medial tibial surface (adjusted OR 2.39, 95% CI: 1.28, 4.48) and medial central weightbearing femoral surface (adjusted OR 2.88, 95% CI: 1.66, 5.00) and each subregion evaluated. There was no significant association between peak KAM and this outcome at any surface or subregion (data not shown). There were no association between baseline KFM and subsequent cartilage thickness loss by either measure of cartilage thickness loss outcome (e.g., adjusted regression coefficient for continuous outcome 0.18, 95% CI: -0.71, 1.08 at the medial tibial surface and 0.65, 95% CI: -0.86, 2.17 at the medial central weightbearing femoral surface).

**Figure 1:**
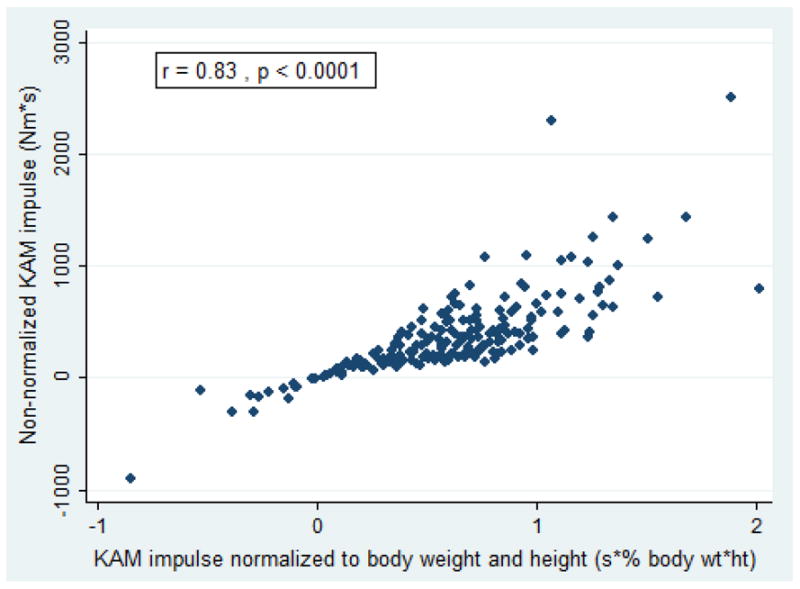
Scatterplot of KAM impulse normalized to body weight and height (s*% body wt*ht) vs. non-normalized KAM impulse (Nm*s) in 197 right knees at baseline. The Spearman correlation coefficient for this association was 0.83 (p<0.0001). For the association between normalized and non-normalized peak KAM values, the correlation coefficient was 0.64 (p<0.0001).

As illustrated in Figure 1, non-normalized KAM parameters correlated strongly with normalized values. In sensitivity analyses, the non-normalized values of the KAM parameter yielded a pattern of results similar to findings using the normalized values.

## Discussion

Greater baseline peak KAM and KAM impulse were each associated with baseline-to-2-year worsening of medial tibiofemoral bone marrow lesions, but not cartilage damage assessed semi-quantitatively. Higher baseline KAM impulse was associated with 2-year medial cartilage thickness loss assessed both as % loss and defined as a threshold of loss exceeding measurement error, whereas peak KAM was related only to % loss. We found evidence of a KAM/cartilage thickness loss relationship in the external and central subregions of the medial femoral and tibial surfaces. In contrast, there was no evidence of an association between baseline peak KFM and any disease progression outcomes.

In a previous longitudinal study of 74 hospital patients with medial knee OA, the risk of baseline-to-6-year radiographic medial OA progression, defined as at least one grade worsening of medial joint space width, increased by 6 fold with every 1-unit (i.e., 1% BW×HT) increase in baseline peak KAM (11). Analysis of a subset of 144 participants pooled from both the interventional and control groups in a 12-month randomized controlled trial of wedge insoles showed that baseline KAM impulse, but not peak KAM, was associated with greater medial tibial cartilage volume loss over 12 months (12). There was no association between KAM parameters and 12-month progression of semi-quantitative measures, i.e., medial tibiofemoral cartilage defects or bone marrow lesions (12). Recently, in 16 individuals with medial knee OA, baseline KAM and KFM were associated with 5-year change in femoral and tibial medial-to-lateral cartilage thickness ratio (13).

Our relatively large (given extensive time and resources required for gait data acquisition and processing) cohort study allowed adjustment for potential confounders that previous studies were unable to fully address. Unlike prior studies, our cohort was recruited predominantly from the community. We evaluated both peak KAM and KAM impulse and semiquantitative and quantitative outcomes. In some instances (medial TF BML progression; ≥ 5% cartilage thickness loss at the medial tibial and femoral surfaces and each subregion), KAM impulse, but not peak KAM, was significantly associated with the outcome. Integrating both load magnitude and duration, KAM impulse may more comprehensively represent cumulative medial load experienced during gait than peak KAM. Compared to peak KAM, KAM impulse has been shown to be more sensitive in discriminating OA disease severity (30) and symptoms (31), and a better predictor of medial-to-lateral ratio of proximal tibial bone mineral density (32). Our findings provide evidence that KAM parameters should be a target in load-modifying interventional trials for persons with medial knee OA.

Using a semi-quantitative approach, we found KAM parameters were associated with bone marrow lesion but not cartilage damage progression. Since baseline presence of bone marrow lesions predicted subsequent site-specific cartilage loss in persons at risk for or with knee OA (33–35), baseline-to-2-year worsening of bone marrow lesion may be a harbinger for future cartilage damage progression. Alternatively, it may require more than 2 years to detect an association with cartilage damage progression using this measure of outcome. Normalized and non-normalized KAM parameters strongly correlated and shared a similar pattern of significant associations with the outcomes.

Contrary to our hypothesis and to a recent report (13), there was no association between baseline peak KFM and any outcome 2 years later. In recent years, the role of peak KFM during gait in medial compartment knee contact load has received more attention. Walter and colleagues (14) showed that the effect of KAM reduction by gait modification did not necessarily guarantee a corresponding decrease in peak medial knee load, likely due to a concurrent deleterious increase of peak KFM. Medial knee load was suggested to be best estimated by a combination of peak KAM and peak KFM (14,15,36). We did not find a link between peak KFM and disease progression, suggesting that a compensatory increase in KFM associated with KAM reduction may not necessarily be deleterious in the structural progression of knee OA. It is plausible that KAM is more strongly associated with medial knee load and that the KFM contribution is less important. Indeed, multivariable regression models for medial knee load indicated that peak KAM had a much greater relative effect on peak medial load than peak KFM (14,36).

In the subregional analysis, KAM parameters were significantly associated with cartilage thickness loss in the medial tibial external and central subregions and central femoral subregion, in keeping with the concept that cartilage in these subregions is subject to greater continuous load in the environment of greater KAM. Although higher peak KFM during gait theoretically may impose greater load on the posterior tibial subregion, our results did not support a negative consequence for the cartilage by 2 years.

The interchangeable use of “KAM” and “medial knee load” has been questioned in recent years (14,15). To further clarify the relationship between KAM and instrumented-implant-measured medial load in a larger sample, Kutzner and colleagues (37) found strong associations between KAM and medial load in early but not in late stance, and that KAM was highly correlated with medial-to-lateral load ratio throughout stance. In a single-subject interventional case report, wearing a medial knee load-modifying variable-stiffness intervention shoe during walking successfully reduced both the first peak KAM and peak medial knee load. KAM reduction strongly predicted reduction in medial load measured *in vivo* (38). Ideally, load-modifying interventions should aim at reducing medial load, not just KAM. Considering the invasive nature of instrumented knee implants and inherent limitations in musculoskeletal models for predicting knee load, KAM may be a sensible alternative. The link between KAM parameters and subsequent disease progression demonstrated in our study confirmed that KAM, although only a determinant of medial knee load, is a reasonable biomechanical target for disease-modifying interventions.

There are several limitations in this study. Follow-up time longer than 2 years may be needed to detect associations between baseline KAM parameters and semi-quantitative cartilage damage progression. Employing an alternative within-grade WORMS scoring method may increase the sensitivity to longitudinal change of cartilage damage (39). Although KFM was not associated with disease progression, alternative novel knee load indices, e.g., the total knee reaction moment (40,41), which represents the magnitude of external knee moments in all three planes, may potentially better capture medial knee load and predict disease progression. Mean BMI of our sample was in the overweight range; results may not be generalizable to a healthy-weight or obese population. Lastly, our sample included knees predominantly with mild OA; findings may differ in knees at a later stage of disease.

As the trajectory and experience of pain in knee OA often does not correspond well to the trajectory of disease progression, future studies should carefully evaluate this outcome. Both quantitative and qualitative aspects of physical activity are important in further understanding the association between KAM and disease progression knee OA. The role of physical activity in the KAM-disease progression relationship should be evaluated in future studies.

In conclusion, in persons with knee OA, greater baseline peak KAM and KAM impulse were each associated with baseline-to-2-year worsening of medial tibiofemoral bone marrow lesions but not cartilage damage assessed semi-quantitatively. Higher baseline KAM impulse predicted 2-year medial cartilage thickness loss assessed quantitatively both as percent loss and as loss defined by a threshold, whereas peak KAM was related to medial cartilage loss only as % loss. There was no evidence of a relationship between baseline peak KFM and any measure of progression. These findings support targeting KAM parameters in an effort to delay medial OA disease progression.

## Figures and Tables

**Table 1:** Peak KAM, KAM impulse, and peak KFM at baseline in knees without and with semiquantitative cartilage damage progression and bone marrow lesion progression, mean (SD) (n = 391 knees from 204 persons)

		Cartilage damage progression at 2-year follow-up			Bone marrow lesion progression at 2-year follow-up		
		Medial tibiofemoral compartment 61/391 (15.6%)#	Medial femoral surface 48/391 (12.3%)	Medial tibial surface 29/391 (7.4%)	Medial tibiofemoral compartment 87/391 (22.3%)	Medial femoral surface 53/391 (13.6%)	Medial tibial surface 48/391 (12.3%)
Peak KAM (% body wt*ht)	Knees without progression	1.66 (0.87)	1.67 (0.86)	1.65 (0.86)	1.59 (0.86)	1.65 (0.87)	1.60 (0.85)
	Knees with progression	1.70 (0.77)	1.68 (0.81)	1.83 (0.76)	1.92 (0.77)	1.77 (0.73)	2.15 (0.71)
KAM impulse (s*% body wt*ht)	Knees without progression	0.60 (0.43)	0.60 (0.43)	0.59 (0.43)	0.55 (0.42)	0.59 (0.44)	0.56 (0.42)
	Knees with progression	0.64 (0.50)	0.64 (0.51)	0.73 (0.51)	0.79 (0.47)	0.70 (0.43)	0.92 (0.45)
Peak KFM (% body wt*ht)	Knees without progression	2.11 (0.86)	2.10 (0.86)	2.10 (0.86)	2.08 (0.89)	2.09 (0.88)	2.10 (0.87)
	Knees with progression	1.98 (0.80)	2.01 (0.81)	1.98 (0.84)	2.13 (0.74)	2.15 (0.71)	2.08 (0.78)

# Percentage of knees (%) with 2-year progression

**Table 2:** Spearman correlations for pairs of continuous variables at baseline (n = 197 right knees)

	Age	K/L grade	Knee alignment (varus is negative)	Gait speed	KAM	KAM impulse	KFM
Age	1.00	0.17	0.12	-0.18	-0.02	-0.02	-0.11
K/L grade		1.00	-0.12	-0.15	0.08	0.15	-0.09
Knee alignment (varus is negative)			1.00	0.03	-0.71	-0.76	-0.23
Gait speed				1.00	0.10	-0.06	0.44
KAM					1.00	0.95	0.15
KAM impulse						1.00	0.05
KFM							1.00

**Table 3:** Association of peak KAM and KAM impulse at baseline with semiquantitative medial tibiofemoral 2-year outcomes: adjusted odds ratios (95% CI) (n = 391 knees from 204 persons)

Baseline predictor variable	Cartilage damage progression			Bone marrow lesion progression		
	Medial tibiofemoral compartment 61/391 ((15.6%)	Medial femoral surface 48/391 (12.3%)	Medial tibial surface 29/391 (7.4%)	Medial tibiofemoral compartment 87/391 (22.3%)	Medial femoral surface 53/391 (13.6%)	Medial tibial surface 48/391 (12.3%)
Peak KAM (% body wt*ht)	0.98 (0.71, 1.34)	0.97 (0.68, 1.37)	1.18 (0.82, 1.72)	1.29 (0.95, 1.74)	1.04 (0.81, 1.35)	**1.52** **(1.09, 2.12)**
KAM impulse (s*% body wt*ht)	0.96 (0.53, 1.73)	0.95 (0.50, 1.81)	1.41 (0.59, 3.36)	**2.20** **(1.12, 4.38)**	1.30 (0.80, 2.12)	**3.29** **(1.46, 7.41)**

The table shows the association between peak KAM and KAM impulse at baseline (independent variables) and medial tibiofemoral cartilage damage progression and bone marrow lesion progression 2-year outcomes (dependent variables, each defined by any worsening of subregion WORMS score), adjusted for gait speed, age, gender, K/L grade, knee pain severity, and medication use. Adjusted odds ratios (OR) and 95% confidence intervals (CI) are presented; 95% CI excluding 1 is significant.

**Table 4:** Association of peak KAM and KAM impulse at baseline with 2-year quantitative medial tibiofemoral cartilage thickness loss (% loss): adjusted regression coefficients (95% CI) (n = 385 knees from 203 persons)

Baseline predictor variable	% Cartilage thickness loss (n=385)						
	Medial tibial surface				Medial central weightbearing femoral surface		
	Whole (n=385)	Central subregion (n=384)	External subregion (n=378)	Posterior subregion (n=385)	Whole (n=384)	Central subregion (n=380)	External subregion (n=373)
Peak KAM (% body wt*ht)	**1.25** **(0.03, 2.48)**	**2.50** **(0.09, 4.91)**	**4.35** **(0.65, 8.06)**	0.88 (-0.14, 1.91)	**2.70** **(0.18, 5.22)**	**4.42** **(0.16, 8.67)**	-3.44 (-17.95, 11.07)
KAM impulse (s*% body wt*ht)	**3.38** **(1.33, 5.42)**	**6.25** **(2.40, 10.10)**	**10.98** **(5.02, 16.94)**	2.07 (-0.06, 4.20)	**7.62** **(4.15, 11.08)**	**12.16** **(5.71, 18.61)**	-13.70 (-60.50, 33.11)

The table shows the association between peak KAM and KAM impulse at baseline (independent variables) and 2-year cartilage thickness loss (dependent variable, % loss as a continuous variable), adjusted for gait speed, age, gender, K/L grade, knee pain severity, and medication use. Adjusted regression coefficients and 95% confidence intervals (CI) are presented; 95% CI excluding 0 is significant.

**Table 5:** Peak KAM, KAM impulse, and peak KFM at baseline in knees without and with quantitative medial cartilage thickness loss (≥ 5%) by surface and subregions, mean (SD) (n = 385 knees from 203 persons)

		Medial tibial surface				Medial central weightbearing femoral surface		
		Whole (74/385) (19.2%)#	Central subregion (103/384) (26.8%)	External subregion (93/378) (24.6%)	Posterior subregion (74/385) (19.2%)	Whole (103/384) (26.8%)	Central subregion (124/380) (32.6%)	External subregion (98/373) (26.3%)
Peak KAM (% body wt*ht)	Knees without progression	1.61 (0.82)	1.60 (0.82)	1.59 (0.82)	1.63 (0.83)	1.56 (0.81)	1.54 (0.83)	1.58 (0.83)
	Knees with progression	1.91 (0.94)	1.85 (0.93)	1.84 (0.91)	1.81 (0.93)	1.95 (0.92)	1.89 (0.85)	1.85 (0.89)
KAM impulse (s*% body wt*ht)	Knees without progression	0.55 (0.39)	0.55 (0.39)	0.54 (0.39)	0.57 (0.40)	0.53 (0.38)	0.51 (0.38)	0.53 (0.39)
	Knees with progression	0.80 (0.56)	0.73 (0.54)	0.74 (0.53)	0.74 (0.56)	0.80 (0.53)	0.78 (0.50)	0.76 (0.52)
Peak KFM (% body wt*ht)	Knees without progression	2.11 (0.87)	2.12 (0.89)	2.11 (0.89)	2.11 (0.88)	2.09 (0.87)	2.10 (0.87)	2.09 (0.85)
	Knees with progression	2.01 (0.81)	2.03 (0.75)	2.07 (0.77)	2.03 (0.76)	2.10 (0.82)	2.10 (0.84)	2.09 (0.88)

# Proportion of knees (%) with 2-year progression
